# Fisetin Rescues the Mice Brains Against D-Galactose-Induced Oxidative Stress, Neuroinflammation and Memory Impairment

**DOI:** 10.3389/fphar.2021.612078

**Published:** 2021-02-25

**Authors:** Sareer Ahmad, Amjad Khan, Waqar Ali, Myeung Hoon Jo, Junsung Park, Muhammad Ikram, Myeong Ok Kim

**Affiliations:** Division of Life Science and Applied Life Science (BK 21 Plus), College of Natural Sciences, Gyeongsang National University, Jinju, South Korea

**Keywords:** d-galactose, fisetin, neurodegeneration, aging model, phytonutrient

## Abstract

Herein, we have evaluated the protective potentials of Fisetin against d-galactose-induced oxidative stress, neuroinflammation, and memory impairment in mice. d-galactose (D-gal) causes neurological impairment by inducing reactive oxygen species (ROS), neuroinflammation, and synaptic dysfunction, whereas fisetin (Fis) is a natural flavonoid having potential antioxidant effects, and has been used against different models of neurodegenerative diseases. Here, the normal mice were injected with D-gal (100 mg/kg/day for 60 days) and fisetin (20 mg/kg/day for 30 days). To elucidate the protective effects of fisetin against d-galactose induced oxidative stress-mediated neuroinflammation, we conducted western blotting, biochemical, behavioral, and immunofluorescence analyses. According to our findings, D-gal induced oxidative stress, neuroinflammation, synaptic dysfunctions, and cognitive impairment. Conversely, Fisetin prevented the D-gal-mediated ROS accumulation, by regulating the endogenous anti-oxidant mechanisms, such as Sirt1/Nrf2 signaling, suppressed the activated *p*-JNK/NF-kB pathway, and its downstream targets, such as inflammatory cytokines. Hence, our results together with the previous reports suggest that Fisetin may be beneficial in age-related neurological disorders.

## Introduction

Aging is a cause of several chronic diseases, including diabetes mellitus, cardiovascular diseases, cancer, and neurological disorders such as Alzheimer’s disease (AD), Parkinson’s disease (PD), and Huntington’s disease (HD) (Khan et al., 2018). Several studies have indicated that elevated ROS level is responsible for different neurodegenerative conditions in various age-associated disorders such as AD, PD, and diabetes (Olanow, 1993; Khan et al., 2019a). The elevated oxidative stress triggers cellular damage to the macromolecules (proteins, lipids, and DNA), which disturbs the physiological functions of the central nervous system (CNS), leading to neurodegeneration (Paradies et al., 2011). Thus, the neurodegeneration caused by elevated oxidative stress could be a therapeutic target to tackle age-related diseases neurodegenerative diseases. To develop neuroprotective strategies against age-related neurological disease, different animal models have been developed. One of the known models is the D-gal injected animal model.

Chronic administration of D-gal induces brain aging and accelerates artificial senescence which is used for different anti-aging pharmacological research (Cui et al., 2006). D-gal is a monosaccharide, which exists throughout the body. At higher concentrations, in the presence of galactose oxidase, it converts to hydrogen peroxide and aldose, causing disposition of a superoxide anion, oxygen-derived free radicals, and cellular damage (Lu et al., 2007). Chronic administration of d-galactose for 2 months induces cognitive and memory impairment through the accumulated ROS, mitochondrial deficits, neuroinflammation, and neurodegeneration (Zhang et al., 2007).

Recently, the use of phytonutrients and medicinal herbs have gained a special interest to treat neurological disorders such as AD (Huang and Mucke, 2012; Shah et al., 2017b). Among the phytonutrients, Fisetin (3,7,3′’,4′’-tetrahydroxyflavone), a natural flavonoid is found in different fruits, such as legumes, mangoes, kiwis, strawberries, grapes, cucumbers, nuts, beans, and onions. Fisetin has shown strong anticarcinogenic, anti-inflammatory, antioxidant, neurotrophic, and neuroprotective effects against different neurodegenerative diseases (Khan et al., 2013; Currais et al., 2014; Echeverry et al., 2015; Maher, 2015).

Here, we explore the underlying neuroprotective mechanism of Fis against D-gal-induced aging in mice. We have targeted the main cell survival mechanisms of the brain, such as silent information regulator transcript-1 (SIRT1), Nuclear factor erythroid 2-related factor 2 (Nrf-2), and Heme oxygenase (HO-1). The Sirt1 is a nicotinamide adenine dinucleotide (NAD)-dependent nuclear histone deacetylase, which is involved in the modulation of several cell survival mechanisms, e.g., regulation of calorie restriction and improving the lifespan, cellular metabolic processes, cells senescence, apoptotic cell death, oxidative stress, neuroinflammation, histones deacetylating and non-histone proteins in the body. The deacetylation by SIRT1 may inhibit the transcriptional activation of NF-kB, and neuroinflammation (Shah et al., 2017a).

The transcription factor Nrf2 encodes antioxidant enzymes, regulates the repair of damaged proteins and organelles, neuroinflammation, and mitochondrial homeostasis. The function of Nrf2 is affected in several neurodegenerative conditions, such as Alzheimer’s disease, Parkinson’s (Khan et al., 2020), and multiple sclerosis. Pharmacological modulators of Nrf2 have shown promising effects in neurodegenerative conditions.

Prion disease is a group of neurodegenerative diseases affecting humans and animal species. The conversion of a non-pathogenic normal cellular protein (PrP^c^) into an abnormal pathogenic form prion protein scrapie (PrP^Sc^), is considered the cause of the disease. The PrP^Sc^ aggregates in the individual’s brain, causing an elevation of oxidative stress, by reducing the expression of Nrf2. And, boosting the level of Nrf2 may reduce the severity of the disease (Shah et al., 2018). Studies have suggested that regulation of Nrf2 may provide an opportunity to delay the progression of this disease (Dinkova-Kostova et al., 2018; Ikram et al., 2019a).

Among the neuroinflammatory factors, Mitogen-Activated Protein Kinases (MAPK), such as *p*-JNK play a critical role in the execution of neuroinflammation. Another factor is the NF-kB, which regulates multiple adaptive and innate immune functions and serves as a pivotal mediator of inflammatory reaction (Liu et al., 2017). The activation of NF-κB has been widely implicated in the normal aging processes, which aggravates the release of inflammatory cytokines and activation of astrocytes and microglial cells, such as ionized calcium-binding adaptor molecule 1 (Iba-1), and GFAP respectively (Ullah et al., 2020b; Kim et al., 2020).

The present study was undertaken to analyze the effects of Fisetin against D-gal-induced elevated ROS, neuroinflammation, and cognitive dysfunction in mice. Herein, we hypothesize that Fisetin may reduce oxidative stress and neuroinflammation by regulating cell survival (SIRT1/Nrf2) and inflammatory (*p*-JNK) mechanisms. Collectively, here we hypothesize that Fisetin may regulate the oxidative stress-mediated neuroinflammation, apoptotic cell death and cognitive dysfunctions in D-gal treated mice.

## Materials and Methods

### Animals Handling, Grouping, and Ethical Approval

The study included wild-type mice (C57BL/6N) having 26–29 g of body weight and 9 weeks of age. The mice were obtained from Samtako Bio (Osan, South Korea). The study was conducted under the approved guidelines of the Institutional Animal Care and Use Committee (IACUC) of the Division of Applied Life Science, Gyeongsang National University, South Korea (approval ID: 125). To acclimatize, all animals (total number of mice = 60, the number of mice per group = 20, and 10 mice for Western blot and 10 for immunofluorescence analysis) were kept for 7 days in the animal house under a 12/12 h light/dark cycle at control temperature (23°C) with 60 ± 10% humidity. The animals were freely provided with food and water.

### Chemicals and Antibodies

Fisetin (Lot#SLBF3913V) and d-galactose (sc-202564) were procured from Sigma-Aldrich Chemical Co. (St. Louis, MO, United States). The drugs were dissolved in 0.1% dimethyl sulfoxide (DMSO) and the final volume was adjusted with normal saline (0.9% saline). The control mice were injected with normal saline.

The antibodies used in the current studies are: SIRT1 (sc-74465), anti-Nrf2 (sc-722), anti-HO-1 (sc-136,961), P-JNK (sc-625), anti-p-NF-κβ (sc-136,548), anti-Iba-1 (sc-32,725),anti-GFAP (sc-33673) anti-interleukin (IL-1β) (sc-32,294), anti-tumor necrosis factor-α (TNF-α) (sc-52,746), NOS-2 (sc-651), anti-Bax (sc-7480) anti-Bcl2 (sc-7382), cleaved Caspase-3 (sc-7272), anti-PARP-1 (sc-8007), anti-PSD-95 (sc-71,933), anti-synaptosomal-associated protein 23 (SNAP-25), and anti-β-actin (sc-47,778) (Santa Cruz Biotechnology, Dallas, TX, United States). The primary antibodies were diluted in 1× TBST (1:1,000), and secondary anti-mouse HRP (horseradish peroxidase) conjugated (Promega Ref# W402) and anti-rabbit HRP conjugated (Promega Ref# W401) were diluted 1:10,000 in 1 M TBST (Promega, Fitchburg, WI, United States); the secondary antibodies (anti-mouse Ref# A11029 and anti-rabbit Ref# 32,732) used in the immunofluorescence studies were diluted in 1:100 in 1 M PBS.

### Mice Grouping and Drugs Administration

**FIGURE 1:**
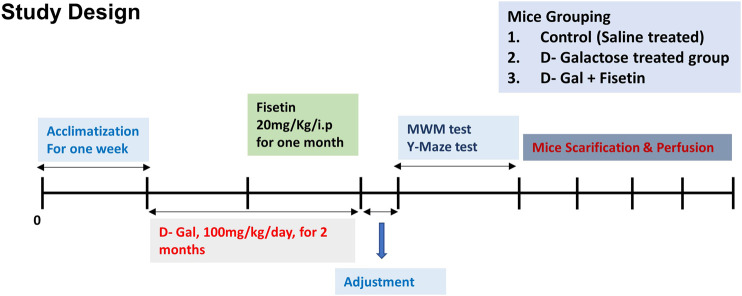
Study design and animal treatments. Figure showing the mice grouping, handling, drug treatment, and experimental design.

The mice were randomly divided into the following three groups 1) Mice injected with saline as a control group. 2) Mice administered with D-gal (100 mg/kg/day i. p for 2 months, one month before, and one month co-treated with Fisetin). 3) Mice administered with D-gal and Fis (20 mg/kg/day i. p for 1 month). The alone group was not considered for the analysis, as no side effects of Fis have been reported previously. The dose of Fis was selected based on previously conducted studies (Rehman et al., 2017). After the completion of the treatment, the behavioral study of the male mice was conducted by using the Y-maze test followed by the Morris water maze (MWM) Figure 1.

### Y-Maze Test

To conduct the behavioral studies, the mice were randomly divided into three groups, and the cages were labeled with numbers (so that the experimenters were unaware of the mice grouping). Y-maze test was conducted to analyze the spatial working memory, as performed previously (Khan et al., 2018; Muhammad et al., 2019). The Y-maze had three arms, having 50 cm in length, 10 cm in width, and 20 cm in height. The mouse was put at the center of the maze and allowed to explore the maze for 8 min (three times), and the arm entries were observed visually. The Spontaneous alternation was defined as the consecutive entry of the mice into the three arms in coinciding triplet sets. The alternation behavior (% age) was calculated as successive triplet sets/(total number of arm entries–2) × 100.

### Morris Water Maze Test

The apparatus used for the MWM is made up of a circular water tank 40 cm in height and 100 cm in diameter. The tank was filled with water (23 ± 1°C) to a depth of 15.5 cm and was made opaque by adding non-toxic white ink. A platform (20 cm in height, 10 cm in diameter) was placed 1 cm below the water surface in one quadrant. The mice were trained for four consecutive days (four trials per day). The latency to reach the hidden platform was calculated for each trial. After completion of the training the probe test was conducted, where the mice were subjected to swim freely in the tank for 1 min (in the absence of platform). Here, the time spent in the target quadrant, and three non-quadrants (right, left, and opposite), and the number of crossings were recorded through a video detective system (SMART, Panlab Harvard Apparatus, United States).

### Protein Extraction From Mice Brains for the Western Blot

After the behavioral studies, the mice were sacrificed and the brains were extracted and the sections (cortex and hippocampus) were carefully dissected, and homogenized in PRO-PREP extraction solution (iNtRON, Seoul South Korea). After homogenization, the brain tissues were centrifuged at 13,000 rpm at 4°C for 24 min, and the supernatants were collected and preserved at (−80°C) for further experimentations.

### Western Blotting

The amount of protein loaded in each well was calculated using the Bio-Rad solution (Bio-Rad protein assay kit, Bio-Rad Laboratories, CA, United States) (Muhammad et al., 2018; Khan et al., 2019a). Equal amounts of proteins 18–20 µg were loaded in the gel under similar experimental conditions. A broad-range prestained protein marker (GangNam STAIN™, iNtRON) was loaded for the determination of the exact molecular weight. To avoid the non-specific binding the membranes were blocked in 5% (w/v) skim milk and incubated with the primary antibodies (overnight at 4°C). The membranes were washed and treated with horseradish peroxidase-conjugate (HRP)-a secondary antibody, and the expressions were detected using an ECL detection reagent, according to the instructions (Amersham, Uppsala, Sweden). The optical densities of the bands were analyzed through ImageJ software.

### Preparation of the Samples for the Immunofluorescence Studies

The mice were perfused transcardially with saline followed by transfusion with ice-cold 4% neutral buffer paraformaldehyde. The brains were fixed in 4% paraformaldehyde for 72 h, followed by incubation in 20% sucrose for 48 h. After that, the brains were fixed in the frozen O.C.T. compound (Tissue-Tek O.C.T. compound medium, Sakura Finetek United States, Inc., Torrance, CA, United States). The 14 μm coronal plane sections were cut using a CM 3050 S Cryostat (Leica, Berlin Germany). The sections were taken on the gelatin-coated slide and used for further experiments.

### Immunofluorescence Staining

The immunofluorescence staining was conducted as described previously (Ikram et al., 2019b; Muhammad et al., 2019). The slides were washed with 0.01 M PBS, followed by incubation for 1 h in 2% normal goat serum and 0.3% Triton X-100 in PBS. After that, the slides were treated with primary antibodies diluted in PBS. After the primary antibody treatment, the slides were treated with appropriate secondary antibodies (TRITC or FITC-labeled) (Santa Cruz Biotechnology, Dallas Texas United States). For the nuclear staining, the slides were incubated with 4′,6-diamidino-2-phenylindole (DAPI). The immunoreaction was visualized using a confocal laser-scanning microscope (Fluoview FV 1000 MPE, Olympus, Tokyo, Japan). Through ImageJ, the relative integrated densities were evaluated among the experimental groups, which sums all of the pixels within a region and gives a total value. And the obtained values were compared among the experimental groups.

### ROS and LPO Assays

The ROS assay was conducted according to the established protocols (Ikram et al., 2019b). The assay is based on the oxidation of 7-dichlorodihydrofluorescein diacetate (DCFH-DA) to 2, 7-dichlorodihydrofluorescein (DCF). In short, the brain tissue homogenates (Cortical and Hippocampal) were used for this assay. The tissue homogenate was diluted in 1 ml of Locke’s buffer (1:20 ratio), 10 ml of DCFH-DA (5 mM), and 0.2 ml of tissue to a final concentration of 5 mg tissue/mL, followed by incubation for 15 min to get a fluorescent DCF. The converted fluorescent DCF was measured using a spectrofluorometer (excitation at 484 nm and emission at 530 nm). A parallel blank was used for background fluorescence (conversion of DCFH-DA in the absence of homogenate). The data has been presented as picomole DCF formed per minute per milligram of the protein. For the evaluation of LPO, the free malondialdehyde (MDA) was analyzed in the brain samples, for which MDA colorimetric/fluorometric kit (Cat #K739–100) was used, a detailed description has been given previously (Badshah et al., 2019).

### Fluoro-Jade B Staining

The Fluoro-Jade B (Burlington, MA, United States, Cat #AG310, Lot #2159662) staining was performed as conducted previously (Ikram et al., 2019b). The slides were dipped in 1% sodium hydroxide and 80% ethanol for 5 min, followed by treatment with 70% ethanol for 2 min. After that, the slides were rinsed with D-water and transferred into a potassium permanganate solution (0.06%) for 10 min, washed with D-water, and kept in 0.1% acetic acid solution and 0.01% Fluoro-Jade B solution for 20 min. After the treatment, the slides were washed with D-water and dried. The sections were covered, and the images were taken using a confocal laser microscope (FV 1000, Olympus, Tokyo, Japan). And, the integrated density was used for the immunofluorescence intensity, for which the ImageJ software (wsr@nih.gov, https://imagej.nih.gov/ij/) was used.

### Evaluations and Statistical Analysis

ImageJ software was used to measure the values for western blot and immunofluorescence analysis. The data has been presented as mean ± SEM (three independent experiments for 20 mice per group: 10 for immunofluorescence and 10 for Western blot). For statistical analysis, we used GraphPad Prism v6 (GraphPad Software), where one-way ANOVA followed by student’s t-test was used to differentiate among the experimental groups. *p* values less than 0.05 was considered to be statistically significant *p < 0.05, **p < 0.01 represent control vs. D-gal treated mice, #*p* < 0.05, ##*p* < 0.01 represent D-gal vs. D-gal + Fis.

## Results

### Effects of Fisetin Against d-Galactose-Induced Oxidative Stress in Mice Brains

**FIGURE 2:**
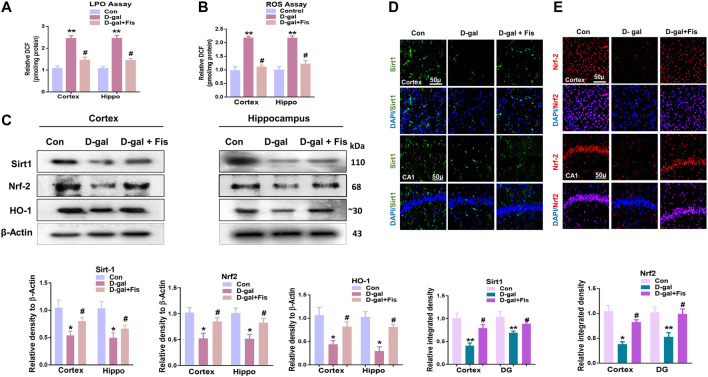
**Fisetin regulates Sirt-1/Nrf2/HO-1 signaling pathway in mice brains (A and B)**. LPO and ROS assays *in vivo*. **(C)**. Western blots results of SIRT1/Nrf2/HO-1 in the experimental groups, with respective bar graphs. β- Actin was used as a loading control **(D and E)**. Immunofluorescence images of SIRT1 and Nrf2 with respective bar graphs Magnifications ×10. Scale bar 50 µm *n* = 10 mice per group for western blot and immunofluorescence analysis, number of experiments = 3. *p < 0.05, **p < 0.01 represent control vs. D-gal treated mice, #*p* < 0.05, ##*p* < 0.01 represent D-gal vs. D-gal + fisetin. LPO: Lipid peroxidation, ROS: Reactive Oxygen Species, Con: Control, D-gal: d-galactose, Fis: Fisetin, Hippo: *Hippocampus*.

To analyze the antioxidant effects of Fisetin, we performed the ROS and LPO assays, which showed that D-gal increased the levels of LPO and ROS, which were partially reduced with the administration of Fisetin (Figures 2A,B). Also, we analyzed the expression of SIRT1/Nrf-2 and HO-1 in the experimental mice brains through western blot. According to our findings, there was a significant downregulation in the expression of SIRT1, Nrf-2, and HO-1 in the D-gal-injected mice brains, compared to the control group. Notably, these markers were upregulated in the D-gal + Fisetin co-treated mice, compared to the D-Gal injected mice (Figure 2C). These results were further confirmed with the immunofluorescence analysis, which showed that Fisetin significantly enhanced the expression of SIRT1 and Nrf-2 in the Fisetin-treated mice brains (Figures 2D,E).

### Protective Effects of Fisetin Against D-Gal-Induced Neuroinflammation in Mice Brains

**FIGURE 3:**
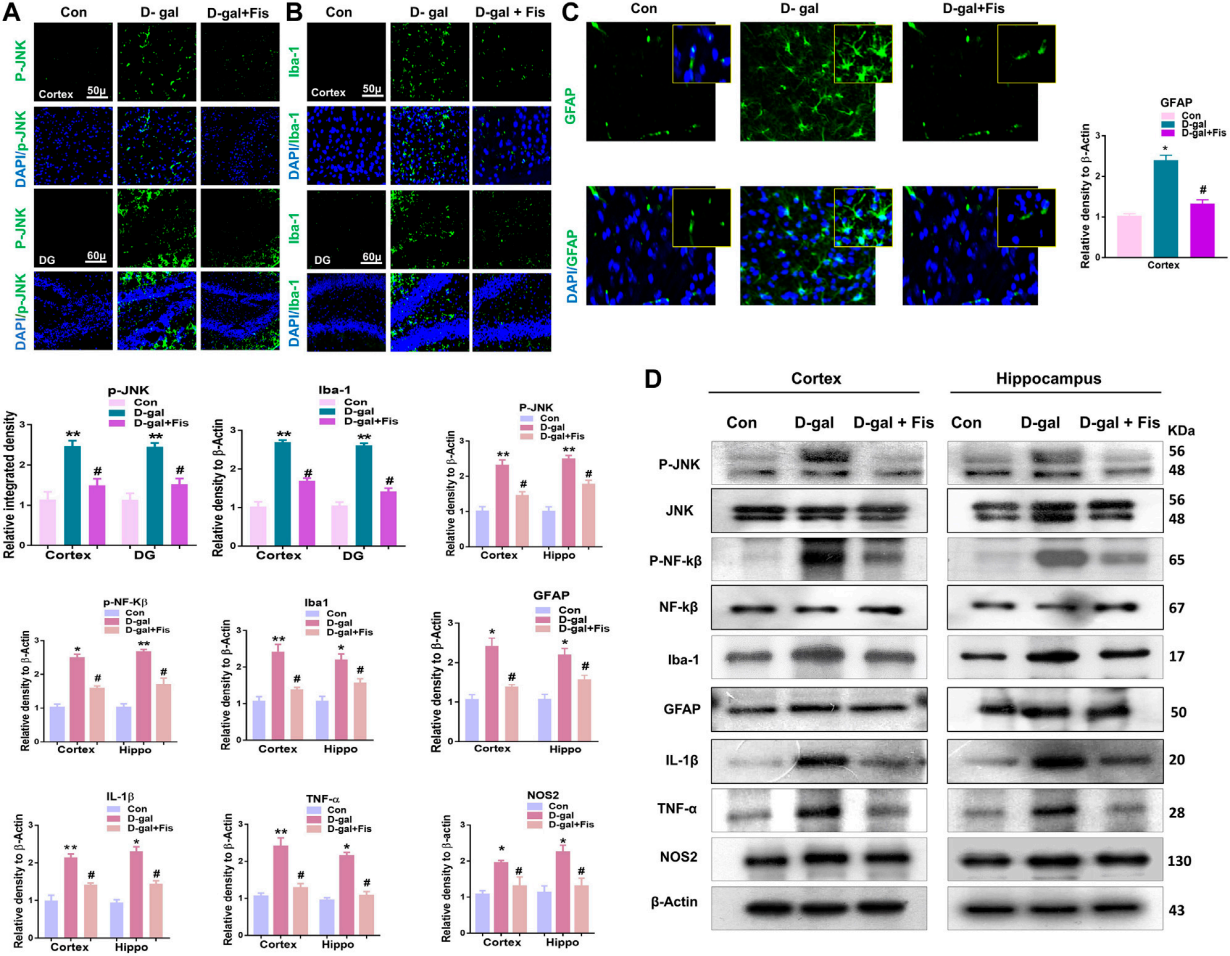
**Fisetin ameliorates the expression of JNK and its associated inflammatory mediators in** **d** **-galactose injected mice (A,B and C)**. Immunofluorescence results of *p*-JNK, Iba-1, and GFAP in the experimental mice’s brains. **(D)**. Western blot results of *p*-JNK, JNK p-NF-Kβ, NF-Kβ, IL-1β, Iba-1, GFAP, TNF-α, and NOS-2 in the experimental mice’s brains. β- Actin was used as a loading control. *n* = 10 mice per group for western blot and immunofluorescence analysis, number of experiments = 3. *p < 0.05, **p < 0.01 represent control vs. D-gal treated mice, #*p* < 0.05, ##*p* < 0.01 represent D-gal vs. D-gal + fisetin. LPO: Lipid peroxidation, ROS: Reactive Oxygen Species, Con: Control, D-gal: d-galactose, Fis: Fisetin, Hippo: *Hippocampus*, DG: Dentate Gyrus.

The elevated ROS level may induce the expression of JNK and NF-kB, which contributes to neuroinflammation in animal models (Rehman et al., 2017). So, we evaluated the expression of *p*-JNK, Iba-1, and GFAP in the experimental groups through the immunofluorescence analysis. According to our findings, there was a significant increase in the expression of *p*-JNK, Iba-1, and GFAP in the D-gal treated mice brains, which was reduced in the D-gal + Fis co-treated groups (Figures 3A–C). Moreover, the western blot results also showed that D-gal-treated mice showed increased expression of p- JNK, JNK p-NF-Kβ, NF-Kβ, Iba-1, GFAP, IL-1β, TNF-α, and NOS-2 in the D-gal-treated mice brains, whereas Fisetin significantly reduced the expression of these markers compared to the D-gal-treated mice (Figure 3D).

### Effects of Fisetin on the Expressions of Apoptotic Markers in the Mice Brains

**FIGURE 4:**
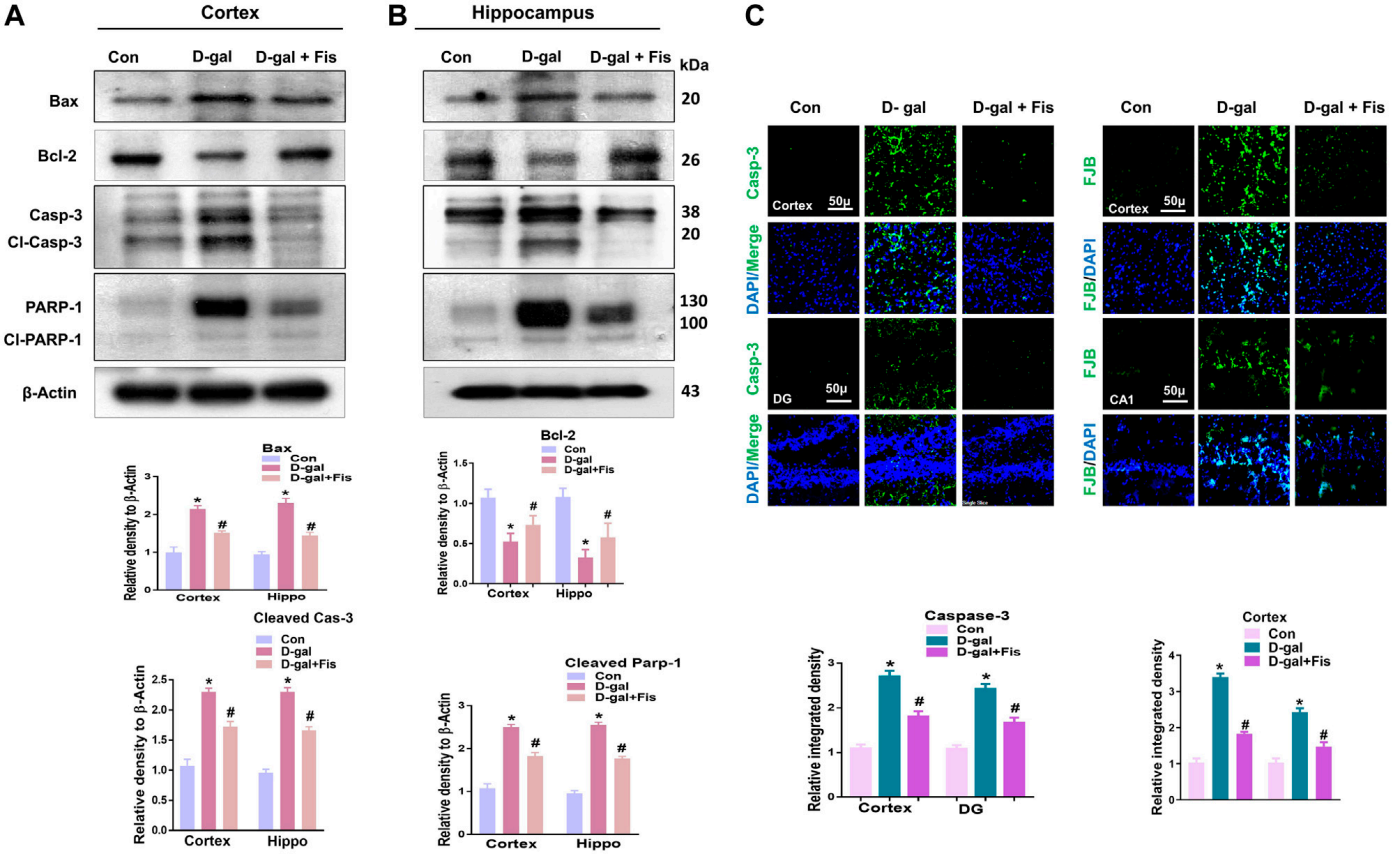
Effects of Fisetin against the apoptotic cell death in the D-gal-treated mice Brains. **(A)**. Western blots results of Bax, Bcl2, cleaved caspase-3, and cleaved PARP-1 in the mice brains **(B and C)** Immunofluorescence results of caspase-3 and flourojade-B in the experimental mice brains. β- Actin was used as a loading control. Values are the means ± SEM from three independent experiments. Magnifications ×10, Scale bar 50 µm *n* = 10 mice per group for western blot and immunofluorescence analysis, number of experiments = 3. *Significantly different from normal mice # significantly different from D-gal-treated mice, respectively; *p < 0.05, **p < 0.01 represent control vs. D-gal treated mice, #*p* < 0.05, ##*p* < 0.01 represent D-gal vs. D-gal + fisetin. Con: Control, D-gal: d-galactose, Fis: Fisetin, Hippo: *Hippocampus*, DG: Dentate Gyrus.

The elevated oxidative stress causes the activation of *p*-JNK which may mediate the apoptotic signalings (Tournier et al., 2000; Liu and Lin, 2005). To show the effects of Fisetin against apoptotic cell death, we analyzed the expressions of cleaved Caspase-3, cleaved PARP-1, and Bax, and Bcl-2 in the D-gal-treated mice brains. Our findings suggested that Fisetin significantly reduced the expression of pro-apoptotic markers (cleaved-Caspase-3. Cleaved-PARP-1 and Bax), and enhanced the expression of anti-apoptotic markers (Bcl-2) in the Fisetin-injected mice brains (Figure 4A). Further, the immunofluorescence results of Caspase-3 showed enhanced activation of Caspase-3 in D-gal-treated mice brains which were reduced in the Fisetin-treated mice brains (Figure 3B). Similarly, the Fluorojad B staining also showed reduced Fluorojad B positive cells in fisetin-treated mice brains, compared to the D-gal injected mice (Figure 3C).

### Effects of Fisetin Against the Synaptic Dysfunctions in the D-Gal Treated Mice

**FIGURE 5:**
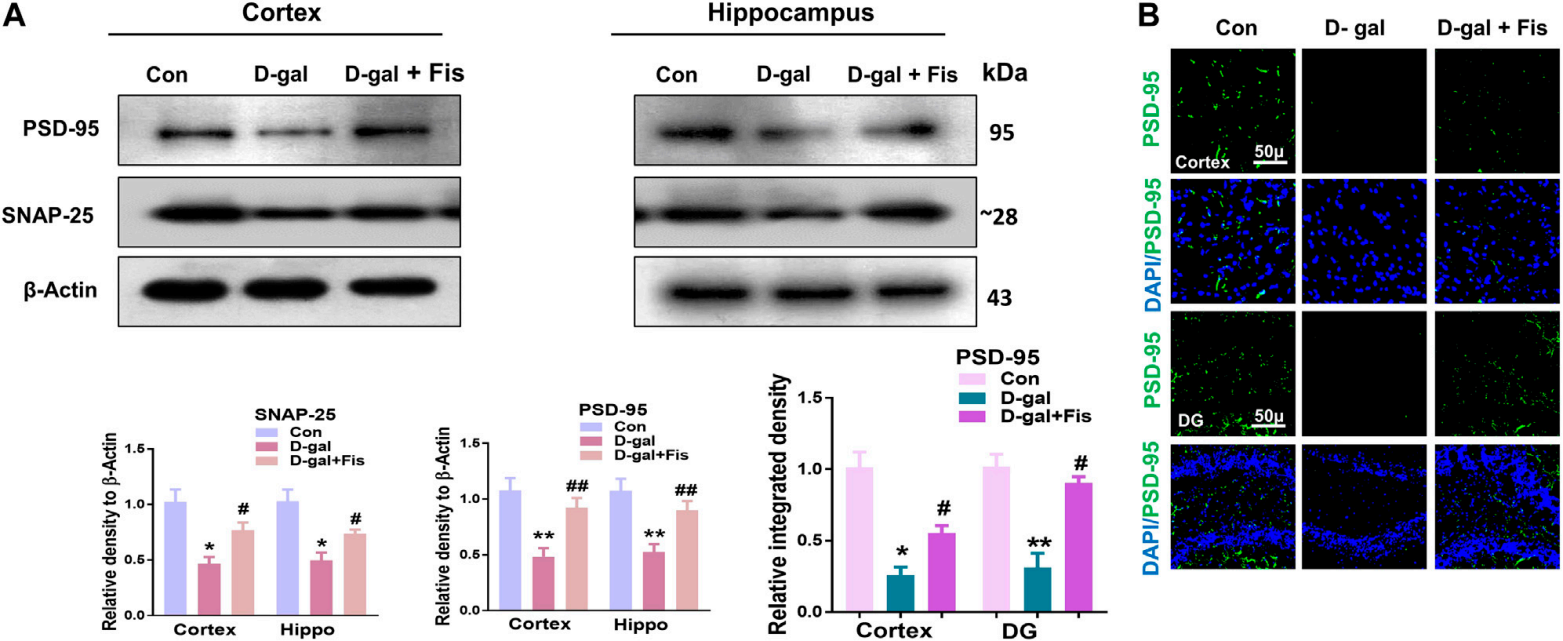
Effects of Fisetin against the synaptic dysfunction in D-gal-treated mice. **(A)**. Western blot resulted in SNAP-25 and PSD-95 in the experimental mice, β- Actin was used as a loading control. **(B)**. Immunofluorescence images of PSD-95 in the cortex and hippocampus of the treated groups. Magnification ×10. Scale bar 50 µm *n* = 10 mice per group for western blot and immunofluorescence analysis, number of experiments = 3. *p < 0.05, **p < 0.01 represent control vs. D-gal treated mice, #*p* < 0.05, ##*p* < 0.01 represent D-gal vs. D-gal + Fis. Con: Control, D-gal: d-galactose, Fis: Fisetin, Hippo: *Hippocampus*, DG: Dentate Gyrus.

Next, we evaluated the expressions of synaptic proteins in the experimental groups. According to our findings, there was a significant downregulation in the expression of synaptic proteins such as SNAP-25 and PSD-95 in the D-gal treated mice compared to the control group, which were upregulated in the Fisetin-treated group (Figure 5A). Moreover, the immunofluorescence analyses also showed reduced expression of PSD-95 in the D-gal-treated mice brains compared to the control group, which were upregulated in the Fisetin-treated mice brains (Figure 5B). Collectively, our findings suggested that Fisetin reversed the D-gal-induced synaptic dysfunction in d-galactose-treated mice brains (Figure 5).

### Effects of Fisetin Against Cognitive Dysfunctions in D-Gal Treated Mice

**FIGURE 6:**
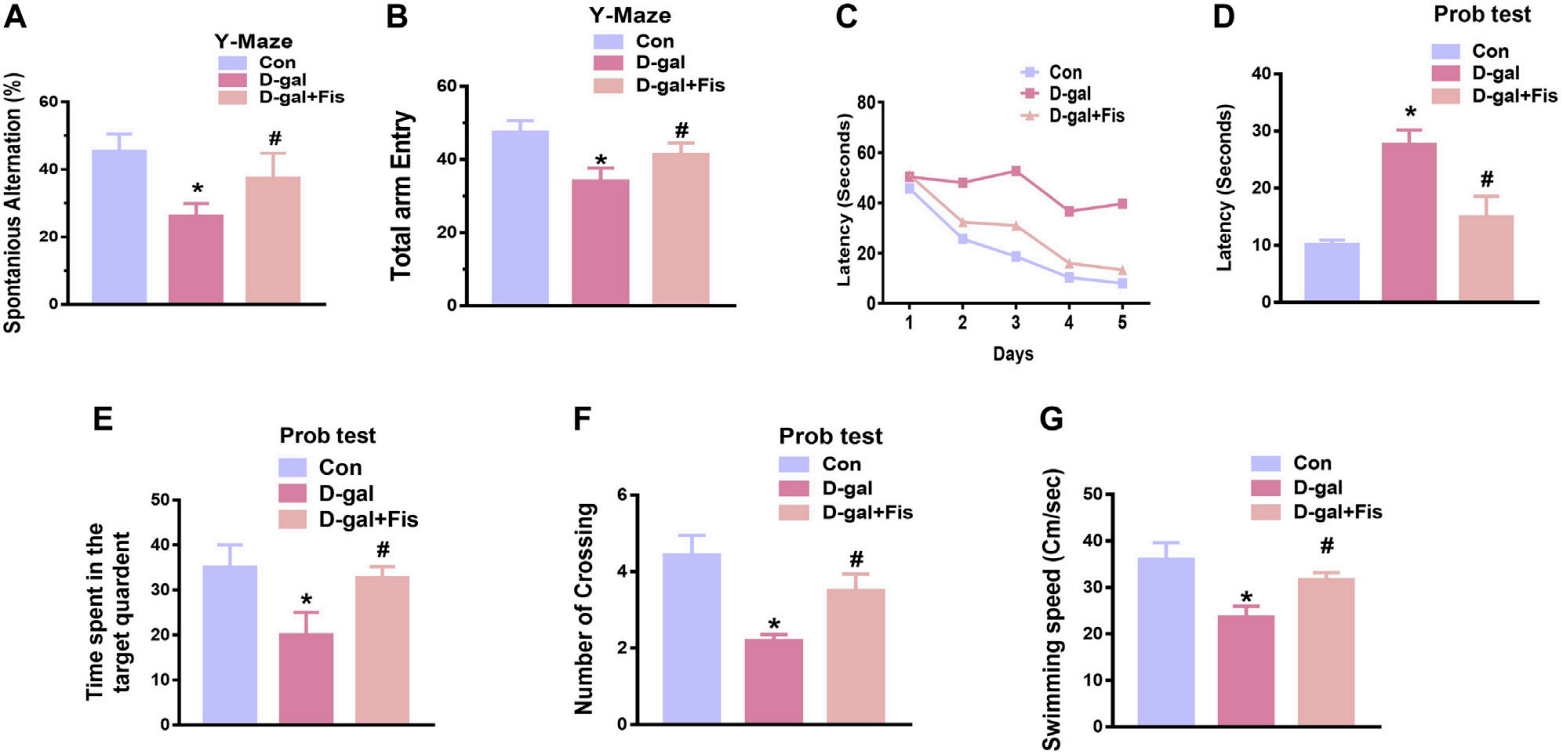
**Fisetin improved cognitive functions in the D-gal-injected mice (A and B)**. Histograms showing the spontaneous alternation behavior (%) and the number of arm entries in the Y-maze test. **(C)**. Latency to reach the hidden platform during the training days. **(D)**. Latency on day fifth. **(E)**. The time spent in the target quadrant during the probe test on day 5. **(F)**. The number of crossings over the hidden platform location during the probe test on day 5. **(G)**. The swimming speed of the mice in the MWM test. *n* = 10 mice per group for behavioral analysis, number of experiments = 3. *p < 0.05 represents control vs. D-gal treated mice, #*p* < 0.05, represents D-gal vs. D-gal + Fis. Con: Control, D-gal: d-galactose, Fis: Fisetin, Hippo: *Hippocampus*.

To assess the effects of fisetin on the cognitive dysfunctions, we performed Y-maze followed by MWM tests. For the evaluation of the spatial working memory, the Y-maze test was conducted. Our results indicated that D-gal-injected mice exhibited less number of spontaneous alternations compared to the saline-treated control mice. Fisetin enhanced the spontaneous alternation behavior (%), showing that Fis restored the spatial working memory of the D-gal-injected mice (Figure 6A). Moreover, the arm entries were also considered, to analyze the effects of Fisetin on the motor performance of mice, which suggested that the number of arm entries was markedly upregulated with the administration of Fisetin, compared to the D-gal treated mice as shown in (Figure 6B).

For the analysis of the memory formation, we performed the MWM test. In the MWM test, the mice were trained to find a submerged hidden platform, if failed to find the platform, the mice were guided to the platform. After the training session, we analyzed the time required to arrive at the hidden platform position. The D-gal-injected mice took more time (increased latency time) to reach the hidden platform compared to the control mice. Interestingly, Fisetin reversed the d-galactose effects and enhanced the memory function, as indicated by the mice taking less time to arrive at the hidden platform compared to the D-gal-treated mice (Figures 6C,D). Furthermore, a probe test was conducted, which demonstrated that Fisetin reversed the d-galactose effects, and increased the number of platform crossings, time spent in the target quadrant (Figures 6E,F). Also, we checked the swimming speed, to show the effects of Fisetin on motor performance. According to our findings, fisetin significantly improved the swimming speed of the mice in the MWM test, compared to the d-galactose injected mice (Figure 6G).

## Discussion

The current study was investigated the antioxidant and neuroprotective effects of Fisetin against d-galactose-induced oxidative stress, neuroinflammation, and memory dysfunctions in mice. Our findings suggested that D-gal induces oxidative stress, neuroinflammation, apoptotic cells, and memory impairment in mice. Interestingly, these effects were markedly reduced with the administration of Fisetin, as confirmed by western blotting, immunofluorescence analysis, and behavioral studies.

Here, we have targeted the four cardinal features of d-galactose-induced aging, i.e., oxidative stress, neuroinflammation, synaptic dysfunction, and memory impairment. Oxidative stress is the result of an imbalance in the pro-oxidant/antioxidant homeostasis leading to the generation of toxic reactive oxygen species (ROS) (Niedzielska et al., 2016). The elevated oxidative stress induces neuroinflammation and neurodegeneration in age-related diseases such as AD (Ikram et al., 2019a) and PD, and other toxin-based animal models of neurodegeneration (Khan et al., 2019a; Badshah et al., 2019).

Several studies have suggested that d-galactose induces aging in animal models, by inducing oxidative stress and neuroinflammation, which aggravate the aging process (Chang et al., 2014). The oxidative stress may be induced by several mechanisms, such as suppression of the endogenous ROS regulators, and inducing lipid peroxidation. As, during aging, there is a significant reduction in the expression of SIRT1, which is vital for different neuronal survival by reducing oxidative stress (Salminen et al., 2013). Also, the cells are endowed with an antioxidant defense mechanism mediated by Nrf2, which activates the transcription of proteins involved in oxidative stress and cytotoxicity (Xue et al., 2012). As activation of Nrf-2 targets several genes such as HO-1, which provide a defense against oxidative stress, neuroinflammation, and neuronal apoptosis, offering a substantial resistance against oxidative stress-induced neurodegeneration (Johnson et al., 2008).

To show the effects of Fisetin against the elevated oxidative stress, we analyzed the expression of SIRT1, Nrf2, and HO-1 in the experimental groups, which suggested that Fisetin significantly regulated the elevated oxidative stress in d-galactose-injected mice brains. As, the downregulation of Sirt1, Nrf-2, and HO-1 in the D-gal treated mice brain, is per the previously conducted studies (Chen et al., 2019). The findings suggest that the d-galactose-induced oxidative stress may be partly due to suppression of the endogenous antioxidant mechanisms, as suggested previously (Ahmad et al., 2019). Interestingly, Fisetin attenuated the oxidative stress, as shown by the ROS and LPO assays.

Another main contributor to the progression of neurodegeneration is inflammation (Ikram et al., 2020), as several studies have shown that D-gal-induces oxidative stress, which activates the inflammatory and apoptotic cell death pathways (Rehman et al., 2019). Several factors are responsible for the induction of neuroinflammation, such as activation of *p*-JNK, p-NF-kβ (Muhammad et al., 2019; Ali et al., 2020), and activation of astrocytes and microglial cells, as components of the innate immune system (Muhammad et al., 2019), which are activated with oxidative stress. Previous studies have suggested that chronic administration of D-gal causes activation of Caspases via *p*-JNK/NF-kβ, which are involved in the neurodegeneration (Lu et al., 2010; Khan et al., 2019a). Nuclear factor-kB, a family of homo- and heterodimeric transcription factors, playing a role in the homeostasis of the various transcription genes in response to different stimuli such as infection, inflammation, and DNA damage-induced oxidative stress (Hayden and Ghosh, 2004), and may result in activation of inflammatory mediators such as IL-1β, TNF-α, and NOS2. Activation of NF-kB has been reported in old-aged mice (Barco and Marie, 2011). In 2013, Rehman et al. suggested that D-gal activates the *p*-JNK/NF-kB pathway (Rehman et al., 2017). Consistent with previous studies, our findings suggested increased expression of *p*-JNK/NF-κB in the d-galactose-injected mice, which were reduced in the Fisetin treated mice. The activation of *p*-JNK/NF-κB elicits other neuroinflammatory mediators and apoptosis. So, we analyzed the expression of IL-1β, TNFα, and NOS2, and other apoptotic markers such as cleaved caspases-3, cleavage PARP-1, Bcl-2, and Bax in the experimental groups, which suggested that fisetin possess strong anti-inflammatory and anti-apoptotic effects. For further confirmation, we used Fluoro-Jade B staining, which showed that, with the administration of Fisetin, there was a significant reversal in the loss of neuronal markers in the D-gal treated mice. The effects of Fisetin against neurodegeneration is in accordance with the previous studies conducted on aluminum (Prakash and Sudhandiran, 2015).

Our findings together with the previously conducted studies on the role of fisetin suggest that Fisetin has pronounced anti-inflammatory effects on neuroinflammation and apoptotic cell death (Ahmad et al., 2019). There is convincing evidence that Fisetin may inhibit the neuroinflammatory mediators, such as suppression of microglia and astrocytes (Chuang et al., 2014), as the activated astrocytes and microglia are the cardinal features of several neurodegenerative diseases (Ullah et al., 2020a) or it may reduce the inflammation by reducing the oxidative stress (Althunibat et al., 2019). The exact role of fisetin against the neuroinflallamtion needed further eluciadation.

Synaptic dysfunction has been extensively reported in age-related diseases such as AD (Ali et al., 2018). However, the exact mechanism by which D-gal causes synaptic dysfunction and neurological disorders remain unclear. Like neuroinflammation, the synaptic dysfunction may be directly triggered with the elevated oxidative stress or due to the activation of the inflammatory mediators, as both of these may affect cognition and synaptic functioning.

## Conclusion

**FIGURE 7:**
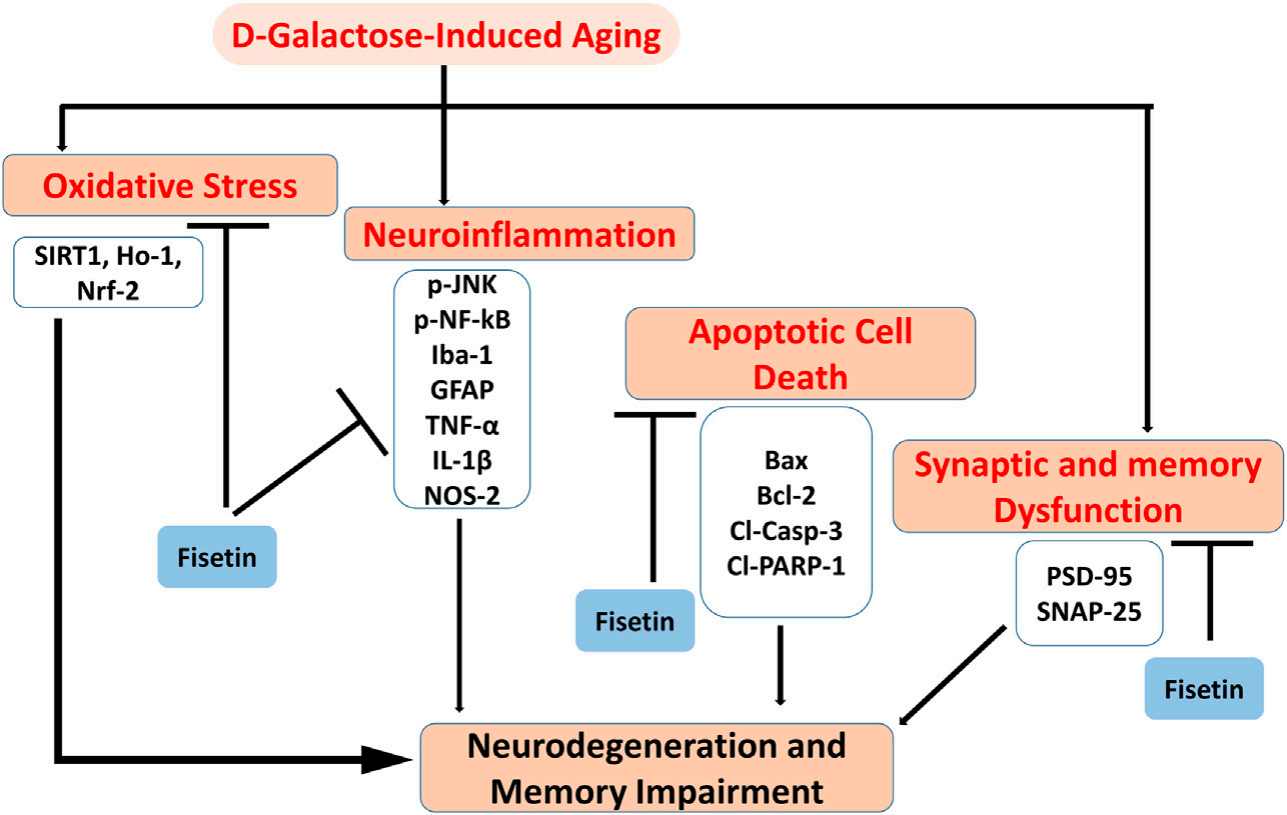
**Graphical Abstract.** The diagram showing that d-galactose causes activation of oxidative stress, neuroinflammation, apoptotic cell death, and synaptic dysfunctions, and cognitive dysfunctions. Interestingly, Fisetin may exert multi-targeted neuroprotective effects by reducing oxidative stress, neuroinflammation, apoptotic cell death, and synaptic dysfunctions.

Collectively our results suggested that Fisetin may attenuate oxidative stress, neuroinflammation, neurodegeneration, and memory impairment in D-gal-treated mice via regulation of SIRT1, Nrf2/HO-1, and *p*-JNK/NF-kB-mediated neuroinflammation. Our study is strongly supporting the previously conducted studies on the role of Fisetin, showing that Fisetin is neuroprotective, by modulating the ionic homeostasis, thereby regulating the vital processes in the neurodegenerative conditions (Singh et al., 2019). Another study also conducted on fisetin, showing that Fisetin abrogated the D-gal induced oxidative stress and neuroinflammation by regulating the inflammatory mediators (IL-1β and TNF-α) and autophagy-related markers (Atg-3 and Beclin-1) (Singh et al., 2018). Previously conducted studies were solely based on the biochemical parameters, no behavioral studies were conducted to support the overall hypotheses. Here, we have conducted detailed synaptic and behavioral studies, which strongly supports the notion that Fisetin may regulate the age-related synaptic and memory impairment in mice. Furthermore, the SIRT1, Nrf2/HO-1 signaling regulated by fisetin is not limited to oxidative stress, and other proteins that affect neuronal apoptosis, inflammatory cytokines, cell survival, and memory performance should also be investigated. As depicted in Figure 7.

## Data Availability

The original contributions presented in the study are included in the article/Supplementary Material, further inquiries can be directed to the corresponding author.
