# Supplementary figures and images for: Fisetin Rescues the Mice Brains Against D-Galactose-Induced Oxidative Stress, Neuroinflammation and Memory Impairment
Source: Front Pharmacol. 2021 Feb 25;12:612078. doi: 10.3389/fphar.2021.612078 (PMC7947859; doi:10.3389/fphar.2021.612078)

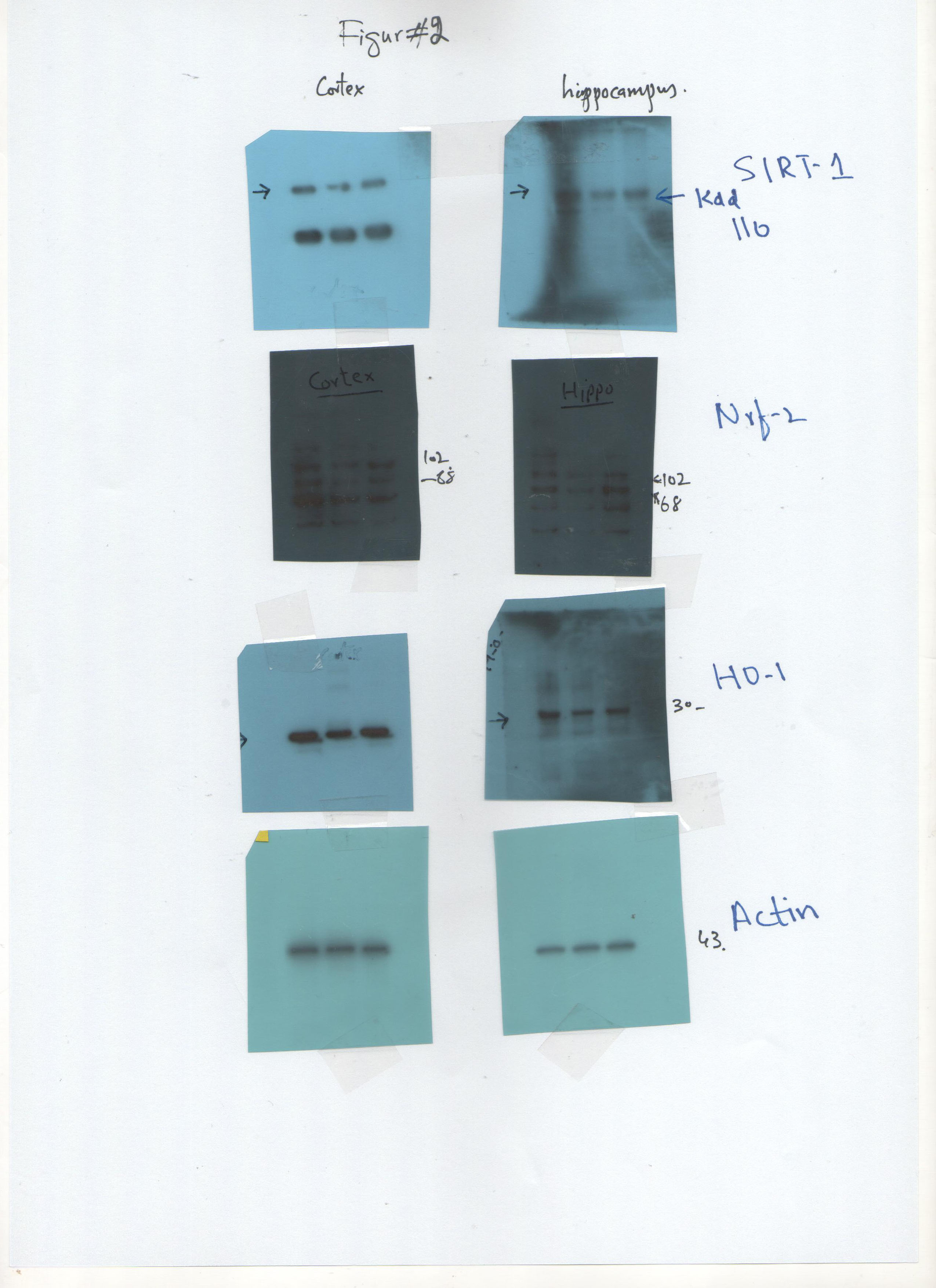

Supplement: Supplementary file 1 [file image1.jpeg]

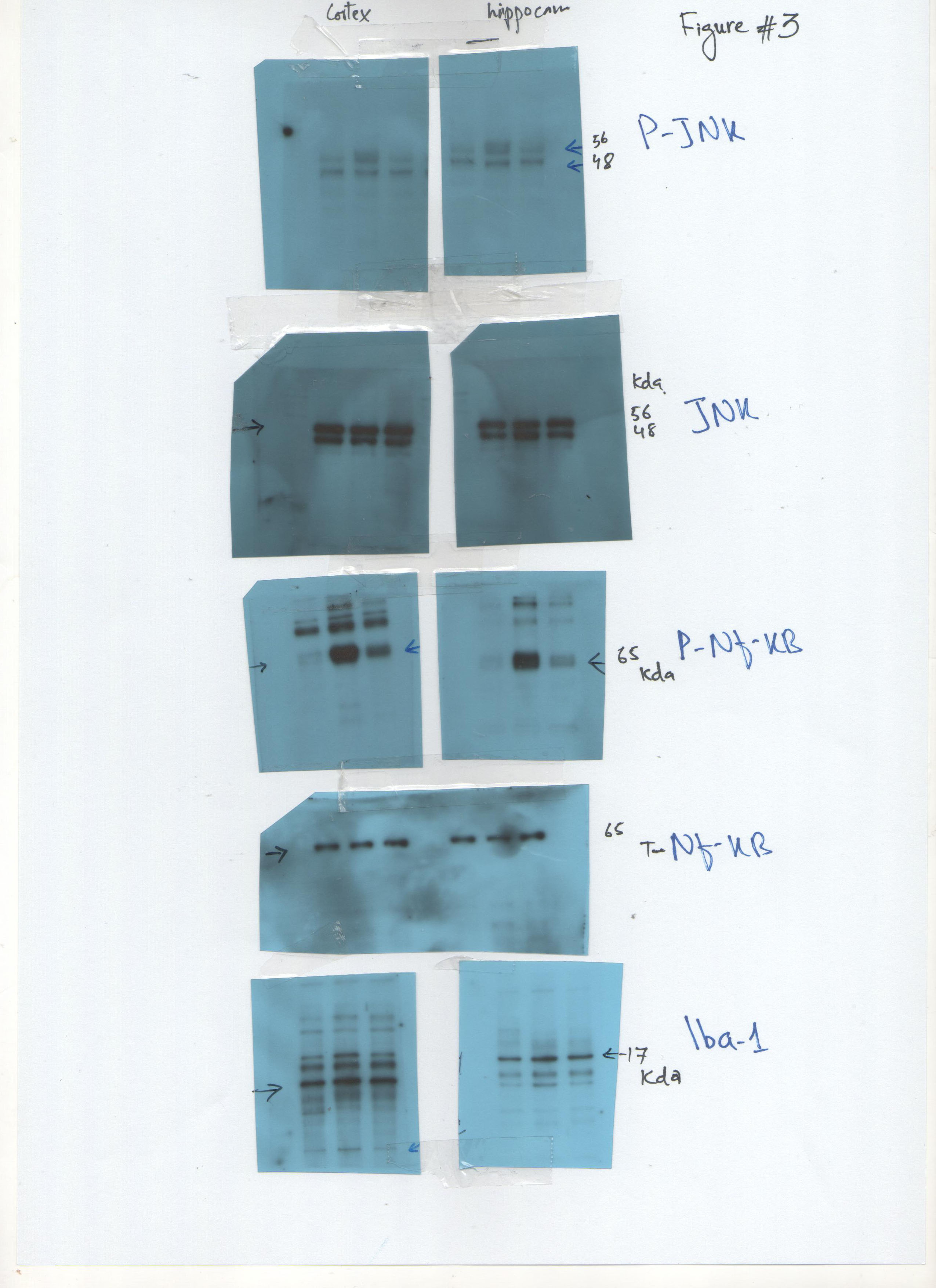

Supplement: Supplementary file 2 [file image2.jpeg]

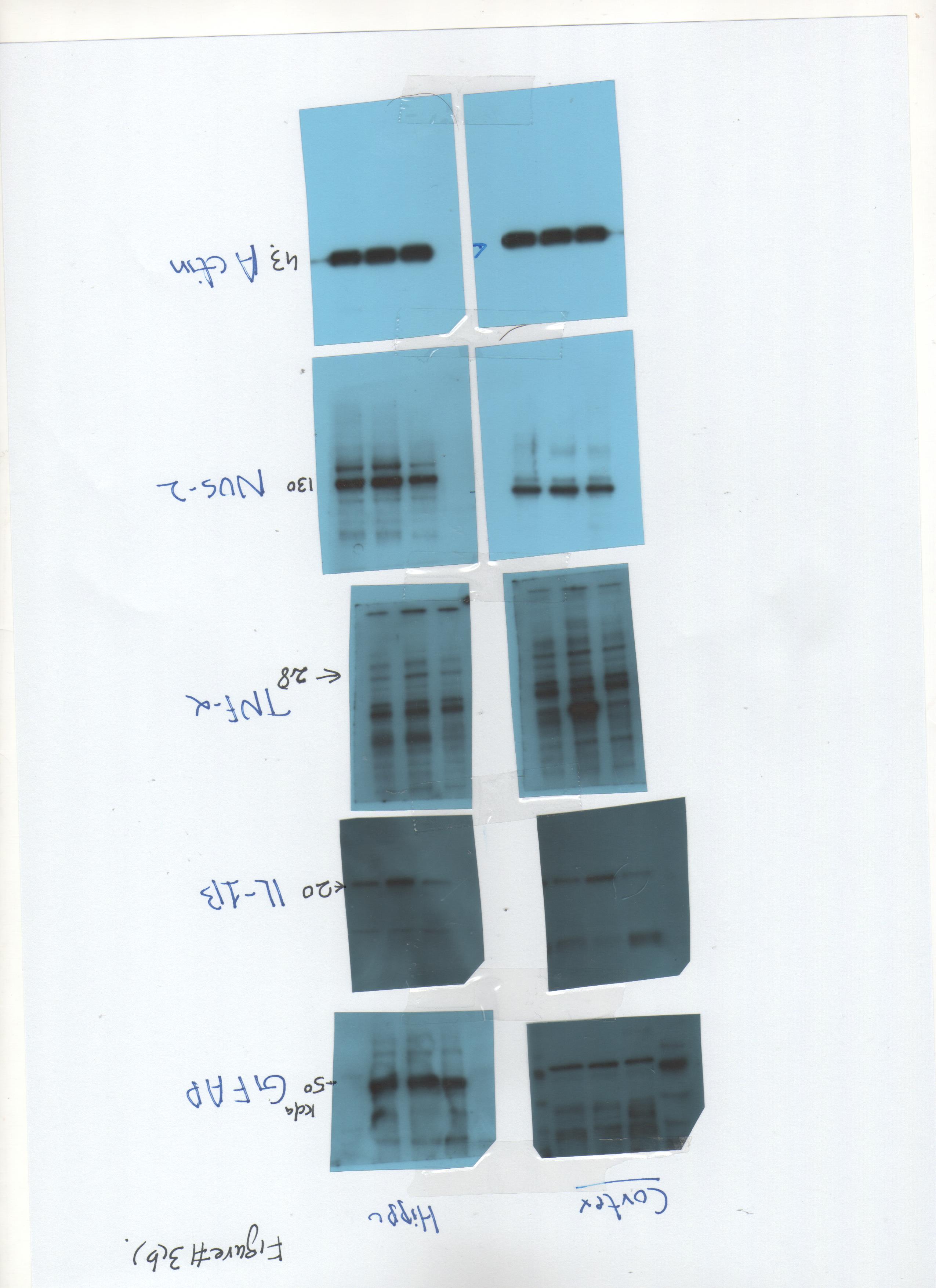

Supplement: Supplementary file 3 [file image3.jpeg]

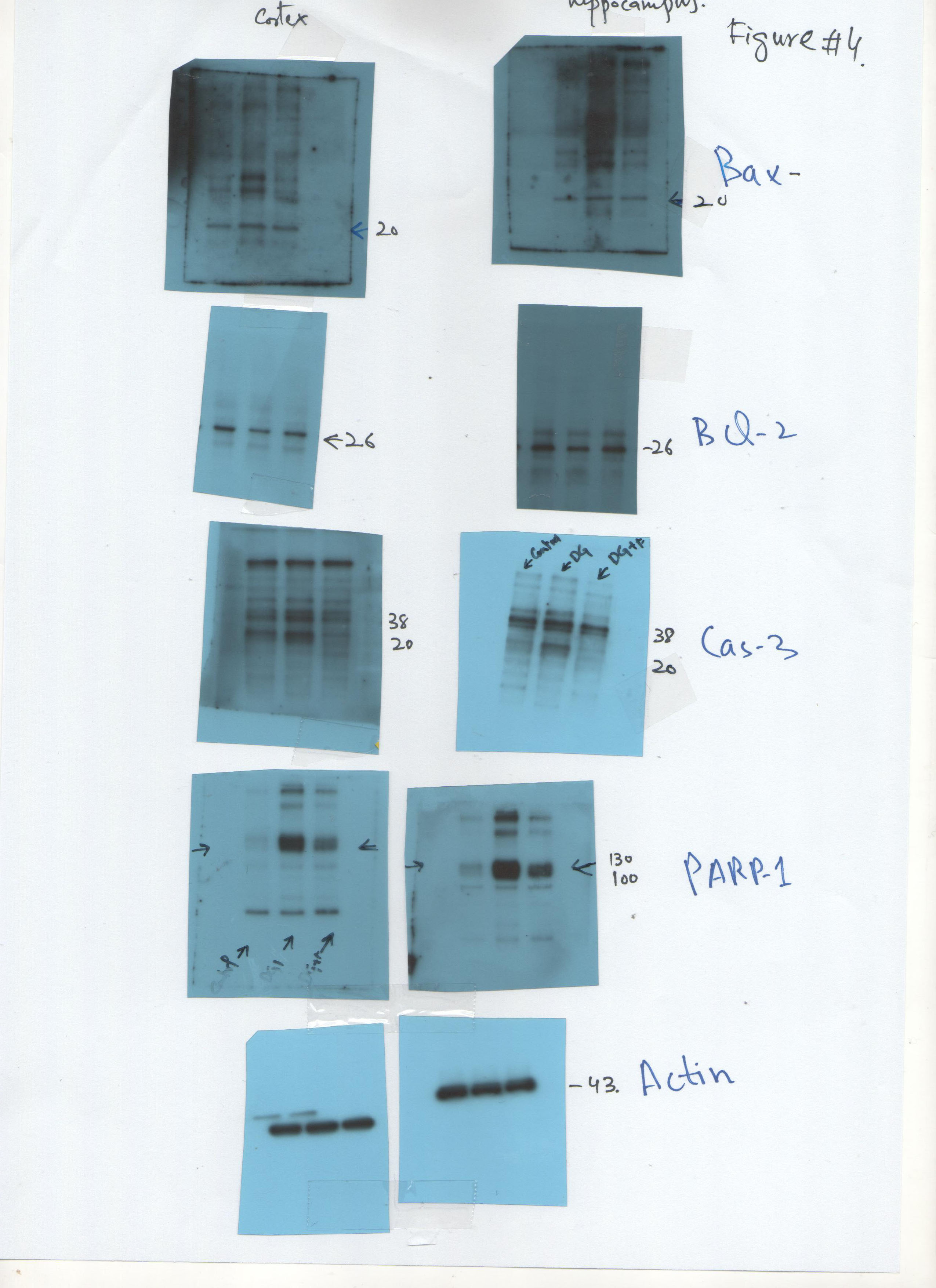

Supplement: Supplementary file 4 [file image4.jpeg]

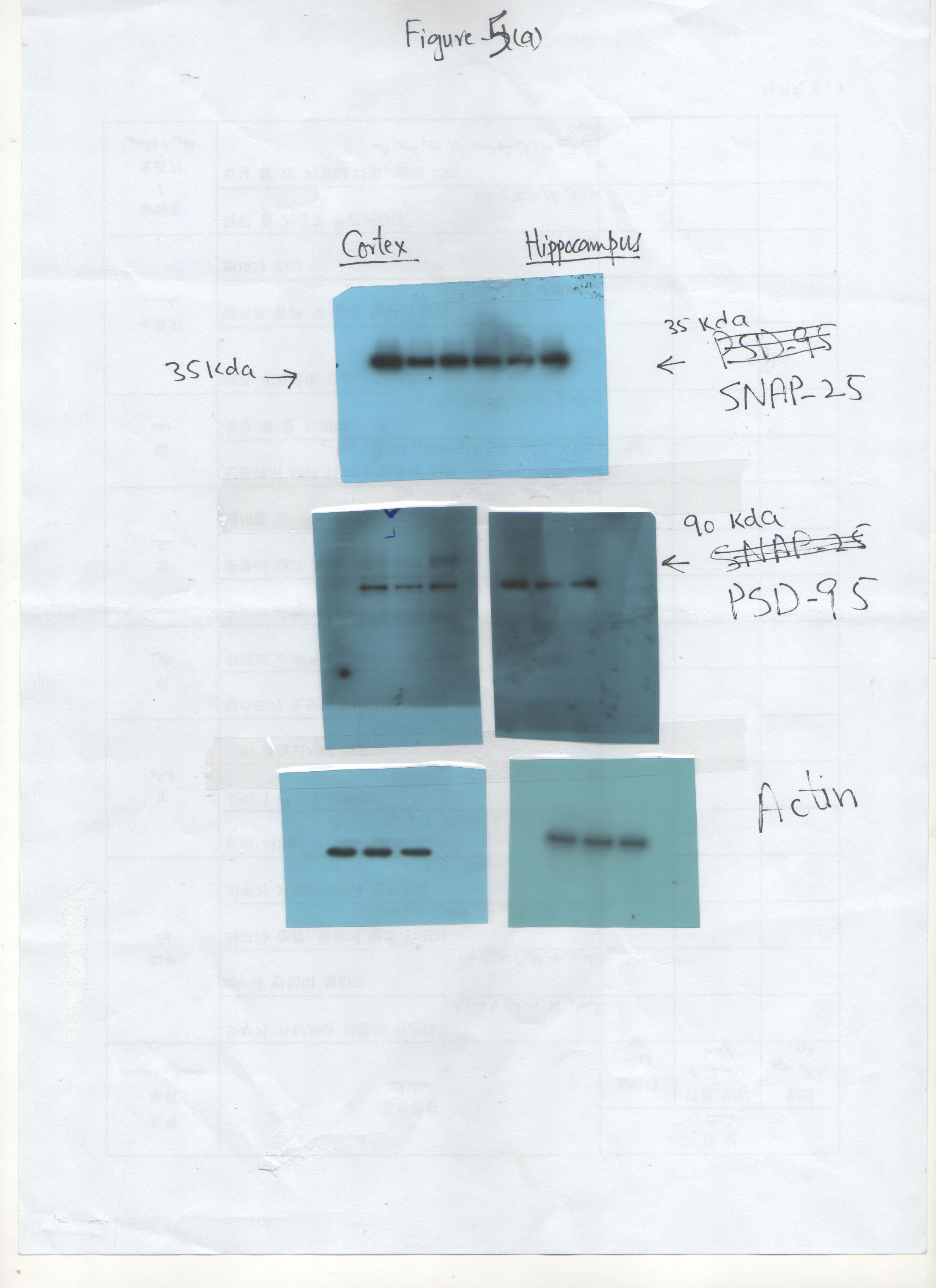

Supplement: Supplementary file 5 [file image5.jpeg]
